# Supplementary material for: Does microcredit for the poverty-alleviated population have an income-increasing effect? Also on the “Raising the Low” effect
Source: PLoS One. 2025 Aug 1;20(8):e0327431. doi: 10.1371/journal.pone.0327431 (PMC12316256; doi:10.1371/journal.pone.0327431)
Supplement: Attachment — Submitted filename: PONE-D-24-05024.pdf [file pone.0327431.s002.pdf]

PLOS ONE

The Income-Increasing Effect of Microcredit for Poverty-Alleviated Population —Also on the "Raising the Low" Effect

--Manuscript Draft--

**Manuscript Number:**	PONE-D-24-05024
**Article Type:**	Research Article
**Full Title:**	The Income-Increasing Effect of Microcredit for Poverty-Alleviated Population —Also on the "Raising the Low" Effect
**Short Title:**	The Income-Increasing Effect of Microcredit for Poverty-Alleviated Population
**Corresponding Author:**	Lu Fan<br>CUEB: Capital University of Economics and Business<br>beijing, CHINA
**Keywords:**	poverty-alleviated population; microcredit; income-increasing effect; raising the low effect
**Abstract:**	Promoting the continuous income increase of the poverty-alleviated population has become one of the key tasks to consolidate the achievements of poverty alleviation, promote rural revitalization and achieve common prosperity. Based on the survey data of 728 farmers in six poverty-alleviated counties (cities) in a province in Northwest China, this paper uses the endogenous transformation regression model to empirically explore the income-increasing effect of microcredit for the poverty-alleviated population, and uses the quantile regression model and OLS regression model to explore the effect of raising the low for the poverty-alleviated population. The results show that : (1) Based on the counterfactual hypothesis, if the farmers who obtain microcredit for poverty-alleviated population are not loaned, their production and operating income will decrease by 3.87 %, that is, microcredit has a significant income-increasing effect, and the income-increasing effect of obtaining microcredit for the poverty-alleviated population on the monitoring objects is greater than that of the poverty-alleviated households. (2) The effect of microcredit for poverty alleviation population on the income increase of low-income farmers with initial endowment is greater than that of high-income farmers with initial endowment, and farmers with low-land initial endowment have a higher income increase effect. The overall efficiency effect on ' low→high ' initial endowment farmers shows a decreasing trend. Therefore, in order to ensure the effectiveness of financial precision assistance, we should promote the microcredit policy of the poverty-alleviated population from the aspects of policy stability and implementation precision.
**Order of Authors:**	Lu Fan
	Yin Liu
	Chong Lin Ma
**Additional Information:**	
**Question**	**Response**
**Financial Disclosure**<br><br>Enter a financial disclosure statement that describes the sources of funding for the work included in this submission. Review the submission guidelines for detailed requirements. View published research articles from *PLOS ONE* for specific examples.	The author(s) received no specific funding for this work.

Powered by Editorial Manager® and ProduXion Manager® from Aries Systems Corporation

This statement is required for submission and **will appear in the published article** if the submission is accepted. Please make sure it is accurate.<br><br>**Funded studies**<br>Enter a statement with the following details:<br>• Initials of the authors who received each award<br>• Grant numbers awarded to each author<br>• The full name of each funder<br>• URL of each funder website<br>• Did the sponsors or funders play any role in the study design, data collection and analysis, decision to publish, or preparation of the manuscript?<br><br>Did you receive funding for this work?	
**Competing Interests**<br><br>Use the instructions below to enter a competing interest statement for this submission. On behalf of all authors, disclose any competing interests that could be perceived to bias this work—acknowledging all financial support and any other relevant financial or non-financial competing interests.<br><br>This statement is required for submission and **will appear in the published article** if the submission is accepted. Please make sure it is accurate and that any funding sources listed in your Funding Information later in the submission form are also declared in your Financial Disclosure statement.<br><br>View published research articles from *PLOS ONE* for specific examples.	The authors have declared that no competing interests exist.

**NO authors have competing interests**<br><br>Enter: *The authors have declared that no competing interests exist.*<br><br>**Authors with competing interests**<br><br>Enter competing interest details beginning with this statement:<br><br>*I have read the journal's policy and the authors of this manuscript have the following competing interests: [insert competing interests here]*<br><br>* typeset	
**Ethics Statement**<br><br>Enter an ethics statement for this submission. This statement is required if the study involved:<br><br>• Human participants<br>• Human specimens or tissue<br>• Vertebrate animals or cephalopods<br>• Vertebrate embryos or tissues<br>• Field research<br><br>Write "N/A" if the submission does not require an ethics statement.<br><br>General guidance is provided below. Consult the submission guidelines for detailed instructions. **Make sure that all information entered here is included in the Methods section of the manuscript.**	N/A,This study did not involve human or animal subjects, It is only a survey of households that have been lifted out of poverty and monitoring households, and thus, no ethical approval was required. The study protocol adhered to the guidelines established by the journal.

Powered by Editorial Manager® and ProduXion Manager® from Aries Systems Corporation

**Format for specific study types**<br><br>**Human Subject Research (involving human participants and/or tissue)**<br>• Give the name of the institutional review board or ethics committee that approved the study<br>• Include the approval number and/or a statement indicating approval of this research<br>• Indicate the form of consent obtained (written/oral) or the reason that consent was not obtained (e.g. the data were analyzed anonymously)<br><br>**Animal Research (involving vertebrate animals, embryos or tissues)**<br>• Provide the name of the Institutional Animal Care and Use Committee (IACUC) or other relevant ethics board that reviewed the study protocol, and indicate whether they approved this research or granted a formal waiver of ethical approval<br>• Include an approval number if one was obtained<br>• If the study involved *non-human primates*, add *additional details* about animal welfare and steps taken to ameliorate suffering<br>• If anesthesia, euthanasia, or any kind of animal sacrifice is part of the study, include briefly which substances and/or methods were applied<br><br>**Field Research**<br><br>Include the following details if this study involves the collection of plant, animal, or other materials from a natural setting:<br>• Field permit number<br>• Name of the institution or relevant body that granted permission	
**Data Availability**<br><br>Authors are required to make all data underlying the findings described fully available, without restriction, and from the time of publication. PLOS allows rare exceptions to address legal and ethical concerns. See the PLOS Data Policy and FAQ for detailed information.	Yes - all data are fully available without restriction

A Data Availability Statement describing where the data can be found is required at submission. Your answers to this question constitute the Data Availability Statement and **will be published in the article**, if accepted.<br><br>**Important:** Stating 'data available on request from the author' is not sufficient. If your data are only available upon request, select 'No' for the first question and explain your exceptional situation in the text box.<br><br>Do the authors confirm that all data underlying the findings described in their manuscript are fully available without restriction?	
**Describe where the data may be found in full sentences. If you are copying our sample text, replace any instances of XXX with the appropriate details.**<br><br>• If the data are **held or will be held in a public repository**, include URLs, accession numbers or DOIs. If this information will only be available after acceptance, indicate this by ticking the box below. For example: *All XXX files are available from the XXX database (accession number(s) XXX, XXX.)*.<br>• If the data are all contained **within the manuscript and/or Supporting Information files**, enter the following: *All relevant data are within the manuscript and its Supporting Information files.*<br>• If neither of these applies but you are able to provide **details of access elsewhere**, with or without limitations, please do so. For example:<br><br>*Data cannot be shared publicly because of [XXX]. Data are available from the XXX Institutional Data Access / Ethics Committee (contact via XXX) for researchers who meet the criteria for access to confidential data.*<br><br>*The data underlying the results presented in the study are available from (include the name of the third party*	All relevant data are within the manuscript and its Supporting Information files.

and contact information or URL).<br>• This text is appropriate if the data are owned by a third party and authors do not have permission to share the data.<br><br>* typeset	
Additional data availability information:	

Manuscript Click here to access/download;Manuscript;renamed_1fbbf.docx

The Income-Increasing Effect of Microcredit for Poverty-Alleviated Population —Also on the "Raising the Low" Effect

Yin Liu [1] ， Lu Fan [2*]， Linchong Ma [3]

1 School of Public Administration, Xinjiang Agricultural University, **2** School of Urban Economics and Public Administration, Capital University of Economics and Business, **3** Marxist College of Xinjiang Agricultural University
[*] 954291004@qq.com

Abstract

Promoting the continuous income increase of the poverty-alleviated population has become one of the key tasks to consolidate the achievements of poverty alleviation, promote rural revitalization and achieve common prosperity. Based on the survey data of 728 farmers in six poverty-alleviated counties (cities) in a province in Northwest China, this paper uses the endogenous transformation regression model to empirically explore the income-increasing effect of microcredit for the poverty-alleviated population, and uses the quantile regression model and OLS regression model to explore the effect of raising the low for the poverty-alleviated population. The results show that : (1) Based on the counterfactual hypothesis, if the farmers who obtain microcredit for poverty-alleviated population are not loaned, their production and operating income will decrease by 3.87 %, that is, microcredit has a significant income-increasing effect, and the income-increasing effect of obtaining microcredit for the poverty-alleviated population on the monitoring objects is greater than that of the poverty-alleviated households. (2) The effect of microcredit for poverty-alleviated population on the income increase of low-income farmers with initial endowment is greater than that of high-income farmers with initial endowment, and farmers with low-land initial endowment have a higher income increase effect. The overall efficiency effect on ' low→high ' initial endowment farmers shows a decreasing trend. Therefore, in order to ensure the effectiveness of financial precision assistance, we should promote the microcredit policy of the poverty-alleviated population from the aspects of policy stability and implementation precision.

1.Introduction

In 2020, the decisive victory of poverty alleviation has been achieved. China has historically eliminated absolute poverty, opened a new journey of rural revitalization, and entered a new historical stage of solidly promoting common prosperity. To promote common prosperity, the most arduous and onerous task is still in the countryside. To realize the common prosperity of farmers in rural areas is a necessary condition and proper meaning for realizing the common prosperity of all the people. Consolidating the achievements of poverty alleviation and preventing large-scale return to poverty are the bottom line requirements for realizing the common prosperity of farmers in rural areas, and promoting the faster growth of income of low-income farmers is the core task of realizing the common prosperity of farmers in rural areas [1]. At the present stage, the monitoring and assistance of the poverty-alleviated population and the promotion of their continuous income increase have become one of the key tasks to consolidate the achievements of poverty alleviation, promote rural revitalization and achieve common prosperity. At the end of 2023, the Central Rural Work Conference emphasized the prevention of poverty return monitoring, the implementation of assistance measures, the enhancement of endogenous motivation, and the continuous consolidation and expansion of poverty alleviation achievements. As a representative financial support measure led by the government, during the period of comprehensively promoting rural revitalization, microcredit for poverty-alleviated population helps poverty-alleviated households and monitoring households to carry out production and development operations, and has become an important engine to enhance the endogenous development momentum of poverty-alleviated population. Continue to further promote the microcredit of poverty-alleviated population, maintain the intensity of credit delivery in poverty-alleviated areas, and fully implement the support policy of microcredit for the poverty-alleviated population to "provide loans to all who need them", which has become an important starting point to promote the effective connection between the achievements of poverty alleviation and rural revitalization, and to achieve common prosperity. Based on this, it is of great practical significance to

study the income increase effect and raising the low effect of microcredit for the poverty -alleviated population.

As an important part of rural finance, micro-credit plays an important role in promoting agricultural and rural development and alleviating rural poverty. Since the implementation of the micro-credit policy, the academic research on the evaluation of its implementation effect has been emerging. At present, for the evaluation of the effect of micro-credit income increase, qualitative description and quantitative analysis are generally used, mainly quantitative methods. This paper mainly uses the propensity score double matching method (PSM), the propensity score double matching difference method (PSM-DID), the instrumental variable quantile regression (IVQR), the two-stage least squares method and the multiple linear regression method to evaluate the income increase effect of microcredit.

For the effect of microcredit income increase, scholars use different methods to draw different conclusions. Some scholars believe that microcredit has effectively improved the accuracy and inclusiveness of financial services for the poor during the period of poverty alleviation. Microcredit can increase the income of the poor, mainly by reducing the access threshold for the poor to obtain formal financial loans, alleviating financial exclusion[2], helping farmers solve the problem of lack of funds, and obtaining more initial funds for further production and operation investment activities [3]. It has improved the production and operation ability of poor households, revitalized various production factors in rural areas, and promoted the large-scale development of local industries on a macro level[4], stimulate the endogenous development momentum of farmers, promote the increase of farmers ' output and income, and have a significant poverty reduction effect[5-6]. Some scholars also believe that although microcredit has significantly increased the operating income of farmers as a whole, it has obvious heterogeneity in the impact on the output of farmers at different income levels[7]. The income effect on high-income farmers and low-income farmers is not significant, but the income effect on middle-income farmers is obvious [8], and there is a strong regional imbalance and spatial dependence [9].

For the poverty-alleviated population microcredit to promote common prosperity. Qi Hongqian and Zhang Jiaxin found that rural inclusive financial policies such as microcredit for poverty-alleviated population can effectively alleviate the relative poverty and mild poverty vulnerability of farmers, and reduce the credit constraints of farmers. It is an important channel for rural inclusive finance to alleviate relative poverty and reduce poverty vulnerability[10]. Through poverty alleviation microcredit, farmers can obtain the funds needed to develop production and operation, increase farmers' net income and family operating income, help promote rural revitalization and solidly promote common prosperity[11]. In the transition period of poverty alleviation and rural revitalization strategy, it is necessary to continue to improve the rural financial poverty alleviation system, give full play to the supporting role of rural finance, and explore the coordination between policy-based financial funds and financial poverty alleviation funds[12].

The above research provides a reference for this article, but there are also shortcomings, mainly as follows : At present, most of the research focuses on the income effect of poverty alleviation microcredit during the period of poverty alleviation, while there are few studies on the income effect of microcredit for the poverty-alleviated population in the period of consolidating the achievements of poverty alleviation and the effective connection of rural revitalization, and there is a lack of microcredit segmentation analysis for different categories of poverty-alleviated population. in the new era, the monitoring object has become the key object of the dynamic monitoring and assistance work to prevent poverty returning. The monitoring object is composed of three types : unstable households, marginal poor households and sudden serious difficulties households. Therefore, based on the existing research and the key work in the new era, this paper focuses on the heterogeneity of the two types of households that have been lifted out of poverty (excluding monitoring objects) and monitoring objects. Based on the micro-survey data of 6 poverty-alleviated counties (cities) in a province in northwest China, covering households who have been lifted out of poverty and monitoring objects, this paper uses the endogenous transformation model to investigate the effect of microcredit on the production and operation income

of the poverty-alleviated population and its differences. At the same time, the quantile regression is used to empirically study the impact of microcredit on the production and operation income of farmers under different initial endowment conditions. By exploring the policy effect of microcredit for the poverty-alleviated population in the new era, it provides empirical evidence for the effectiveness of financial assistance, and provides a policy basis for further guiding rural financial precision assistance and improving the efficiency of financial funds in China.

2.Theoretical analysis and model construction

2.1 Theoretical Analysis and Research Hypothesis

Referring to the research of Wu Benjian et al.[13] and Weng Chen et al.[3], a theoretical model of the impact of microcredit on farmers ' production and operation income is constructed. It is assumed that the production and operation income of farmers is affected by two factors of production : capital and labor. The production function of farmers is as follows :

$$Y = AK^{\alpha} L^{1-\alpha} \qquad (0<\alpha<1) \tag{1}$$

Among them, Y is the income of production and operation, A represents the current level of production technology of farmers, K represents the amount of capital input, and L represents the amount of labor input.

By increasing the amount of capital, the net profit Π obtained by farmers engaged in production and operation activities can be expressed as :

$$\Pi = AK^{\alpha} L^{1-\alpha} - rK \qquad (0<r<1) \tag{2}$$

In Equation (2), r represents the depreciation rate of agricultural assets. The first-order partial derivative of the net profit of production and operation can obtain the optimal capital investment to maximize the profit of farmers :

$$K^{*} = (r/\alpha A)^{1/(\alpha-1)} L \tag{3}$$

Further transformation of Eq. (3) can be obtained：

$$L = K^{*} (r/\alpha A)^{1/(1-\alpha)} \tag{4}$$

Substituting Eq. (4) into Eq. (2), the maximum profit function of farmers can be obtained :

$$\Pi^* = (1/\alpha - 1)rK^* \tag{5}$$

Under the condition of financial support policy, the " two exemptions and one subsidy " microcredit for poverty-alleviated population is used to develop production and operation. It is assumed that the capital input K of farmers engaged in production and operation is composed of two parts, namely, self-owned capital O and poverty-alleviated population microcredit capital M. The proportion of self-owned capital O invested in production and operation is ζ , $\zeta \in [0,1]$; The proportion of poverty-alleviated population microcredit M invested in production and operation is ψ , $\psi \in [0,1]$. Therefore, the capital investment of farmers to carry out production and operation meets the following conditions :

$$K=\zeta O+\psi M \tag{6}$$

On the basis of Eq. (5) and Eq. (6), through the partial derivatives of microcredit for poverty-alleviated population, the impact of microcredit on the net profit of farmers ' production is as follows :

$$\frac{\partial \Pi}{\partial M} = \frac{\partial \Pi}{\partial K} \times \frac{\partial K}{\partial M} = (1/\alpha - 1)\psi r \tag{7}$$

In 2021, the China Banking and Insurance Regulatory Commission, the Ministry of Finance, the People 's Bank of China, and the National Rural Revitalization Bureau jointly issued the " Notice on In-depth and Solid Microcredit Work for Poverty Alleviation Population in the Transition Period, " which clearly stipulates that lenders must adhere to household borrowing, household use, and household repayment, and accurately use it for lenders to develop production and conduct operations. Therefore, when lenders use microcredit for the poverty-alleviated population, they must be used to develop production and carry out operations. When farmers obtain microcredit for the poverty-alleviated population, with the increase of capital factor input, the net income brought by capital factor is increasing. At the same time, compared with the traditional microcredit, the microcredit of the poverty-alleviated population is

completely subsidized by the government, coupled with a more relaxed risk compensation mechanism, which eliminates the debt pressure of the borrower to a certain extent, and has a positive effect on encouraging the borrower to develop production and conduct business. Therefore, based on this, the following hypothesis is proposed :

Hypothesis 1: Compared with farmers who do not obtain microcredit for the poverty-alleviated population, obtaining microcredit for the poverty-alleviated population can promote the increase of their production and operation income. That is, microcredit for the poverty-alleviated population has an income-increasing effect.

The precise implementation of the microcredit policy for the poverty-alleviated population should take into account the initial resource endowment level of farmers. Under the goal of common prosperity, the focus of rural revitalization has shifted from eliminating absolute poverty to alleviating relative poverty. At this stage, how to improve the endogenous development momentum of the poverty-alleviated population and how to achieve the goals of ' raising the low ' and ' expanding the middle ' has become an important task to alleviate relative poverty. In fact, microcredit for poverty-alleviated population mainly helps low-income groups with poor resource endowments to solve financing difficulties [14]. No matter during the period of poverty alleviation or the period of comprehensively promoting rural revitalization, microcredit has played a ' hematopoietic ' role in promoting the production and operation of poverty-alleviated households and monitoring objects. However, in the new era, some poverty-alleviated households and monitoring objects have leaped from low-income groups to middle-income groups through long-term assistance and self-development, and their own resource endowments have been at a high level. In theory, the marginal effect of credit capital input on increasing farmers' income is decreasing [15]. Therefore, for poverty-alleviated households and monitoring objects with poor resource endowments, the higher the income of credit capital input to promote farmers ' production and operation, and for poverty-alleviated households and monitoring objects with higher resource endowments, the effect of credit capital investment on increasing farmers' income is relatively small. That is to say, the microcredit policy for the poverty-

alleviated population has a greater income-increasing effect on the lower-income poverty-alleviated households and monitoring objects engaged in production and operation. Therefore, the following hypothesis is proposed :

Hypothesis 2: There are differences in the income effect of microcredit for poverty-alleviated population on farmers with different initial endowments, and the income effect on farmers with low initial endowments is higher than that on farmers with high initial endowments. That is, the microcredit for the poverty-alleviated population has a ' raising the low ' effect.

2.2 Model construction

2.2.1 Endogenous transformation regression model (ESR)

Whether farmers can obtain microcredit for poverty-alleviated population and the income of their production and operation is also constrained by their own resource endowments, such as the number of family labor, land resources, industrial base, social capital and so on. At the same time, there are also unobservable factors that may affect farmers ' access to microcredit for poverty-alleviated population and the income of production and operation, such as cognitive ability, learning ability, support strength, etc. These unobservable factors will lead to sample selection bias and endogenous problems. There may be a problem of ' simultaneous decision-making ' between the loans decision-making of microcredit for the poverty-alleviated population and the income effect it brings. If models are established separately for estimation, it may also lead to inaccurate research conclusions due to selective bias and endogenous problems caused by unobservable factors [16]. Although the propensity score matching method can solve the problem of sample bias and variable endogeneity, it cannot solve the sample selection bias caused by unobservable factors [17]. The endogenous transformation regression model can simultaneously deal with the impact of observable and unobservable factors on farmers' decision-making to obtain microcredit for the poverty-alleviated population. By building a counterfactual framework, the conditional expectations of the production and operation income of farmers who obtain and do not obtain microcredit for the poverty-alleviated population are analyzed, and then the average treatment effect of farmers' access to microcredit for the poverty-alleviated

population on production and operation income is further analyzed, revealing the real impact of access to microcredit for the poverty-alleviated population on farmers ' production and operation income.

The basic idea of the ESR model is to evaluate it in two stages. The first stage is the behavioral decision equation, that is, to estimate the influencing factors of whether farmers have access to microcredit for the poverty-alleviated population; the second stage is the result equation, that is, the production and operation income of the ' loaned ' and ' non-loaned ' groups of farmers is estimated to study the differences in the production and operation income of the loan households under different circumstances.

The behavioral decision-making equation of whether to obtain microcredit for the poverty-alleviated population is constructed as follows:

$$S_i^* = \pi_i Z_i + \theta_i I_i + \mu_i \quad (8)$$

In the formula, S_i^* represents the latent variable of whether the farmer i obtains the microcredit of the poverty-alleviated population. When $S_i = 1$, it means that the farmer obtains the microcredit of the poverty-alleviated population. When $S_i = 0$, it means that the farmer doesn't obtain the microcredit of the poverty-alleviated population. Z_i represents the exogenous explanatory variable that affects whether the farmer obtains the microcredit of the poverty-alleviated population. I_i represents the identification variable, π_i 、 θ_i represents the coefficient to be estimated, and μ_i represents the random disturbance term.

Therefore, the decision equation of farmers ' production and operation income can be expressed as :

$$Y_{1i} = X_{1i}\beta_1 + \sigma_{1\mu}\lambda_{1i} + \varepsilon_{1i} \quad (S_i = 1) \quad (9)$$

$$Y_{0i} = X_{0i}\beta_0 + \sigma_{0\mu}\lambda_{oi} + \varepsilon_{0i} \quad (S_i = 0) \quad (10)$$

Eq. (9) represents the estimation equation of the production and operation income of the farmers who have obtained the microcredit for poverty-alleviated population, and Eq. (10) represents the estimation equation of the production and operation income of the farmers who have not obtained the microcredit for poverty-alleviated population. Y_{1i} And Y_{0i} respectively represent the production and operation income of the farmers who

have obtained and have not obtained the microcredit for poverty-alleviated population, X_{1i} and X_{0i} respectively represent the factor variables that affect the production and operation income of the farmers in the two situations, ε_{1i} and ε_{0i} represent the random disturbance term, β_1、β_0 represent the coefficient to be estimated. In order to further solve the problem of self-selection bias caused by unobservable variables, the inverse Mills ratioλ_{1i}、$\lambda_{0i}$and its covariance $\sigma_{1u} = cov(\mu_i, \varepsilon_{1i})$ 、 $\sigma_{0\mu} = cov(\mu_i, \varepsilon_{0i})$ are introduced, and the simultaneous estimation of Eq. (8) to Eq. (9) is carried out by using the complete information maximum likelihood method.

By comparing the production and operating income expectations of whether or not to obtain microcredit for the poverty-alleviated population under the factual and counterfactual scenarios, the average processing effect of whether or not to obtain microcredit for the poverty-alleviated population on the production and operating income of farmers is obtained. The estimation process is as follows:

The expected value of the production and operation income of the farmers who have obtained microcredit for the poverty-alleviated population :

$$E[Y_{1i} \mid S_i = 1] = X_{1i}\beta_1 + \sigma_{1\mu}\lambda_{1i} \quad (11)$$

The expected value of production and operation income of farmers who have not obtained the microcredit for poverty-alleviated population：

$$E[Y_{0i}|S_i = 0] = X_{0i}\beta_0 + \sigma_{0\mu}\lambda_{0i} \quad (12)$$

In the counterfactual situation, the expected value of the production and operation income of the farmers who have obtained the microcredit for poverty-alleviated population in the non-acquisition situation :

$$E[Y_{0i} \mid S_i = 1] = X_{1i}\beta_0 + \sigma_{0\mu}\lambda_{1i} \quad (13)$$

The expected value of production and operation income of farmers who have obtained microcredit for poverty-alleviated population in the context of access :

$$E[Y_{1i} \mid S_i = 0] = X_{0i}\beta_1 + \sigma_{1\mu}\lambda_{0i} \quad (14)$$

Through Eq. (11) and Eq. (13), the average treatment effect of farmers ' actual access to the production and operation income of microcredit for the poverty-alleviated population is :

$$ATT_i = E[Y_{1i} \mid S_i = 1] - E[Y_{0i} \mid S_i = 1] = X_{1i}(\beta_1 - \beta_0) + (\sigma_{1\mu} - \sigma_{0\mu})\lambda_{1i} \quad (15)$$

Similarly, through Eq.(12) and Eq. (14), the average treatment effect of the production and operation income of the farmers who actually have not obtained the microcredit for the poverty-alleviated population is :

$$ATU_i = E[Y_{1i} \mid S_i = 0] - E[Y_{0i} \mid S_i = 0] = X_{0i}(\beta_1 - \beta_0) + (\sigma_{1\mu} - \sigma_{0\mu})\lambda_{0i} \quad (16)$$

2.2.2 Quantile regression model

Based on the analysis of the effect of the production and operation income brought by the microcredit for the poverty-alleviated population. this paper draws on the research ideas of Huo Yu et al. [18], and uses the quantile regression model to further estimate the impact of obtaining microcredit for poverty-alleviated populations on the production and operating income of farmers with different initial endowments. Taking the income of farmers ' production and operation (natural logarithm) as the explained variable, whether or not farmers obtain microcredit for the poverty-alleviated population and the characteristics of individual, family, production and operation as the explanatory variables, the following quantile regression model is established:

$$Y_\tau(Y \mid S) = \chi_\tau + \phi_\tau S_i + \omega_\tau X_i + \upsilon_\tau \quad (17)$$

In Eq. (17), $Y_\tau(Y|S)$indicates the production and operation income of farmers in the τ quantile, S indicates the access to microcredit for the poverty-alleviated population, i indicates the i-th farmer, X_i indicates the factors affecting the production and operation income of farmers in addition to the microcredit for the poverty-alleviated population, $\chi_\tau$indicates the constant term, ϕ_τ、ω_τ indicates the coefficient to be estimated, and υ_τ indicates the random disturbance term.

3.Data sources and statistical description of variables

3.1 Data sources

The data in this paper are derived from the sample survey data of farmers in six poverty-alleviated counties (cities) in a province in northwest China from December

25th,2022 to January 10th,2023. This area is one of the ' three states and three districts ' regions with great difficulty and scale of poverty alleviation during the period of poverty alleviation in China. It is typical to study the income effect of microcredit for poverty-alleviated population in this region under the background of comprehensively promoting rural revitalization. A total of 1350 questionnaires were collected. According to the policy, the borrowers of microcredit for the poverty-alleviated population must be poverty-alleviated households and monitoring objects, and must be used for the development of production and operation. Therefore, this paper excludes general households and farmers without production and operation income, and the effective sample is 728. Among them, 324 households have been lifted out of poverty (excluding households with sudden severe difficulties and unstable poverty alleviation), and 404 households have been monitored (including 52 households with sudden severe difficulties, 180 households with unstable poverty alleviation, and 172 households with marginal poverty-prone households). Households that have been lifted out of poverty refer to the original file card households with higher income levels and no risk of returning to poverty at the current stage. The monitoring object refers to low-income households that have experienced a sharp increase in expenditure or a sharp decrease in income due to illness, sudden accidents and other reasons since 2019, and at the time there was a risk of returning to poverty. This study is only a survey of households that have been lifted out of poverty and monitoring households, and both have the oral consent of the respondents. It did not involve human or animal subjects, thus, no ethical approval was required.

3.2 Variable definition and statistical characteristics

Explained variables. This paper selects the per capita production and operation income of the family as the explained variable. In the survey, the sample farmers were asked about the household operating income from planting, breeding, business, etc.in the whole year of 2022. According to the actual living population of the family, the per capita operating income of the household was calculated and the natural logarithm was taken as the explanatory variable of this article.

Core explanatory variables. In essence, this paper mainly discusses the income effect of microcredit for the poverty-alleviated population, so the ' whether to obtain microcredit for the poverty-alleviated population ' in the questionnaire is taken as the core explanatory variable, and this paper only studies the poverty-alleviated households and monitoring objects who meet the qualification of microcredit for the poverty-alleviated population and have developed production and operation.

Control variables. This paper controls the variables that may affect the income of farmers' production and operation, including the variables of household head characteristics (age, gender, education level), family characteristics variables (actual population, proportion of the elderly, proportion of young adults, proportion of disability, proportion of subsistence allowances), production and operation characteristics variables (land transfer in, land transfer out, family social network, skill training, distance from village to county).

Instrumental variables. Based on the selection of instrumental variables by Huo Yu et al. [18] and He Jing et al. [19], considering that the acquisition of microcredit for the poverty-alleviated population will be affected by the ' peer effect ', that is, it may be related to the behavior imitation among farmers of similar ages in the same village. So this article is grouped according to the village and age (40 years old and below, (40 years old, 50 years old], (50 years old, 60 years old], over 60 years old), and calculates the average level of farmers of similar age groups in the same village to obtain microcredit for the poverty-alleviated population as an instrumental variable, and in the extreme case where the average level is 0 or 1, this paper replaces it with the whole sample mean. Theoretically, there is a strong correlation between farmers ' access to microcredit for the poverty-alleviated population and the access to micro-credit for the poverty-alleviated population by farmers of similar ages in the same village, but the average level will not have a direct impact on farmers ' production and operation income, so it meets the requirements of exogenous and correlation of instrumental variables.

Table 1 is the definition and assignment of all variables in this paper. It can be seen from Table 1 that the production and operation income of farmers who obtain microcredit for poverty-alleviated population is higher, which indicates that obtaining

microcredit for poverty-alleviated population may significantly increase the production and operation income level of farmers. In terms of other variables, the farmers who have obtained microcredit for poverty-alleviated people show significantly higher levels of household head characteristics such as gender and education level, the characteristics of families such as the actual population and the proportion of young and middle-aged people, and the characteristics of production and operation such as social networks and skills training. In addition, farmers who do not obtain microcredit for the poverty-alleviated population show a significant high level of household head and family characteristics such as age, proportion of the elderly, proportion of disability, proportion of subsistence allowances, etc. Although Table 1 intuitively reflects that there are significant differences in the mean values of some variables under whether or not to obtain microcredit for poverty-alleviated population, it does not indicate that these differences are caused by whether or not to obtain microcredit for p poverty-alleviated population. In order to accurately demonstrate the income effect of microcredit for the poverty-alleviated population, it is also necessary to fully consider the selective bias caused by the ' self-selection ' of the sample. Therefore, the following uses a more scientific endogenous conversion regression model for empirical analysis.

Table 1. Variable definition, descriptive statistics and mean difference test

	**variable**	**Variable definition and assignment**	**Unloaned households （N=269）**	**Loaned households （N=459）**	**Mean difference**
**Explained variable**	Producing and operating income (Yuan)	In 2022, the per capita production and operation income of households such as planting, breeding and doing business will be	4478.524	4812.399	-333.875
	The natural logarithm of production and operation income	In order to weaken the influence of extreme values, the natural logarithm of per capita production and operation income is taken.	7.794	8.018	-0.224***
**Core explanatory variables**	Microcredit for poverty- alleviated population	The micro-credit for the poverty-alleviated population has been obtained = 1 ; unacquired population microcredit = 0	0	1	-
	Sexuality	Male = 1 ; female = 0	0.803	0.895	-0.092***

**Householder characteristic variable**	Age	The actual age of the head of household	55.357	49.031	6.326***
	Standard of culture	Illiterate and semi-illiterate = 1 ; primary school = 2 ; junior high school = 3 ; high school / technical secondary school = 4 ; university and above = 5	2.312	2.449	-0.137***
**Family characteristic variables**	The actual population	The actual resident population (including the population who live together or have close economic ties).	3.993	4.586	-0.593***
	Proportion of elderly	Number of people aged 60 and above / actual population	0.241	0.07	0.17***
	Proportion of young adults	Number of persons aged 16-59 years / actual population	0.486	0.577	-0.091***
	Proportion of disability	Number of disabled people / actual population	0.064	0.034	0.031***
	Proportion of low-income households	Number of subsistence allowances / actual population	0.491	0.38	0.111***
**Production and operation characteristic variables**	Agricultural land transfer-out	Land transfer = 1, no land transfer = 0	0.112	0.083	0.029
	Agricultural land transfer-into	Land transfer out = 1, no land transfer out = 0	0.071	0.052	0.018
	Social networking	The natural logarithm of household wedding and funeral expenses in 2022	2.168	2.836	-0.668***
	Competence training	Have participated in skill training = 1, have not participated in skill training = 0	0.145	0.27	-0.125***
	Distance	Distance from the village committee to the county (km)	34	37.98	-3.98
**instrumental variable**	Average microcredit access rate	The average level of farmers of similar age groups in the same village to obtain microcredit for the poverty-alleviated population, and the extreme situation with an average level of 0 or 1 is replaced by the full sample average.	0.538	0.656	-0.118***

Note : *, * *, * * * are expressed at a significant level of 10 %, 5 %, 1 %, respectively, the same below.

4.Empirical Test and Result Analysis

4.1 The estimated results of the impact of obtaining microcredit for the poverty-alleviated population on the income of farmers' production and operation.

Table 2 is the estimation results of the ESR model for the impact of obtaining microcredit for the poverty-alleviated population on farmers ' production and operation income. From Table 2, we can see that the correlation coefficient of the error term of the simultaneous estimation of the microcredit of the poverty-alleviated population and the income of the farmers ' production and operation income, ρ1 is significant at the statistical level of 1%, indicating that there is indeed a self-selection bias in the sample. If not corrected, it will lead to biased estimation results. ρ1 is negative, indicating that the production and operation income of farmers who obtain microcredit for poverty-alleviated population is higher than that of random individuals in the sample farmers. This conclusion shows that obtaining microcredit for poverty alleviation can improve the production and operation income of farmers. In addition, the two-stage equation independence LR test in the model rejects the null assumption that the decision-making equation and the result equation are independent at the 1% level, and the Wald test of goodness of fit is significant at the level of 1%, indicating that the endogenous transformation regression model is reasonable. In the loan decision equation of Table 2, the instrumental variable is significantly positive at the statistical level of 1% for obtaining microcredit for the poverty-alleviated population. In order to further verify the effectiveness of the instrumental variable, referring to the research ideas of Li Jiahui and Lu Qian [16], in the case of introducing control variables, OLS regression is carried out with the situation of microcredit access to the poverty-alleviated population and instrumental variable as independent variables, and the production and operation income of farmers as the dependent variable. The results show that the instrumental variable has no significant impact on the production and operation income of farmers. In addition, this study also uses the instrumental variable method (IV-2SLS) for empirical testing. The results show that the first stage F value is 16.36, which is greater than the empirical value of 10, indicating that the instrumental variable is not a weak instrumental variable. Therefore, it can be considered that the instrumental variables selected in this study are effective.

4.1.1 The estimation results of the influencing factors of farmers' access to microcredit for poverty-alleviated population

Among the family characteristic variables, the estimated coefficient of the proportion of the elderly in the loan decision equation is − 0.762, and it is significant at the statistical level of 5%, indicating that the higher proportion of the elderly in the family will significantly reduce the probability of farmers obtaining microcredit for the poverty-alleviated population. The possible reason is that the higher the proportion of the elderly, the less the labor force in the family, and the limited labor force can' t further expand the scale of production and operation, resulting in a relatively small demand for funds to expand the scale of production and operation. Therefore, the households that have been lifted out of poverty and monitoring objects with heavy pension burden will reduce the probability of obtaining microcredit for the poverty-alleviated population. In the characteristics of production and operation, the estimated coefficient of social network in the loan decision equation is 0.0407, which is significant at the statistical level of 1 %, indicating that the rich social network of the family will significantly increase the probability of farmers obtaining microcredit for poverty-alleviated population. The possible reason is that the situation of family social network reflects the social intensity of farmers to a certain extent. The richer the social network, the stronger the social interaction, showing the closer the relationship between farmers. In the process of production and operation of farmers, strong social relations can strengthen their initial endowments of production and operation, such as obtaining labor, machinery, market information and technology needed for production and operation through social relations. At this time, farmers have the need to continue to expand the scale of production and operation, resulting in an increase in the demand for funds to expand the scale of production and operation. Therefore, the richer the social network, the more likely the households that have been lifted out of poverty and monitoring objects will obtain microcredit for the poverty-alleviated population. This is consistent with the research conclusion of Yin et al. [20].

4.1.2 The estimated results of the impact of obtaining microcredit for the poverty-alleviated population on the income of farmers' production and operation.

In the family characteristic variables, the actual population has a significant negative impact on the production and operation income of farmers who obtain microcredit for poverty-alleviated population and those who do not obtain. Generally speaking, the more the actual population of the family, the more the labor force may be, and the rural labor force tends to spontaneously transfer to the non-agricultural sector with higher remuneration, resulting in a decrease in the labor input engaged in agricultural production and operation, which reduces the production and operation income of the loaned and non-loaned families. The proportion of the elderly and the proportion of the disabled have a significant positive impact on the production and operation income of the farmers who obtain the microcredit of the poverty-alleviated population. Families with a high proportion of the elderly or the disabled will generally have the adult labor force to take care of the elderly or the disabled. Although the burden of such families is heavy, the adult labor force still has the ability to develop production and operation. Therefore, with the help of microcredit for the poverty-alleviated population, it can stimulate such families to expand the scale of production and operation, and then increase the production and operation income. The proportion of subsistence allowances has a significant negative impact on the production and operation income of farmers who obtain and do not obtain microcredit for the poverty-alleviated population. Under the precise assistance policy, the higher the proportion of subsistence allowances, the less the family's labor force on behalf of the family, and the less the labor force required for the development of production and operation. Therefore, regardless of whether they are loaned or not, the proportion of subsistence allowances has a negative impact on their production and operation income. In terms of production and operation characteristics, skill training has a significant negative impact on the production and operation income of farmers who obtain microcredit for the poverty-alleviated population. Farmers who participate in skill training not only help to improve their own skill level, but also broaden their knowledge in the training process. And skill training in the region mainly focuses on employment-oriented e-commerce, beauty salons, brick and tile workers, planting and breeding, etc. Therefore, skill training has greatly promoted the transfer of local rural labor to higher-yielding non-agricultural

sectors with higher returns. So skill training has a significant negative impact on the production and operation income of loaned households. Distance has a significant positive impact on the production and operation income of farmers who have obtained and have not obtained microcredit for poverty-alleviated population. The villages closer to the county have significant advantages in transportation, service industry, factory and other aspects. The more employment opportunities the farmers have, the less investment they will make in the development of production and operation. vice versa. Therefore, the farther away from the county, the higher the income of production and operation.

Table 2. ESR model estimation results of the impact of microcredit on farmers' production and operation income

Variable	Loan decision equation	Result equation	
		Loaned	Unloaned
Sexuality	0.212<br>(0.137)	0.122<br>(0.167)	-0.128<br>(0.164)
Age	-0.00289<br>(0.00572)	0.00568<br>(0.00672)	0.00805<br>(0.00671)
Atandard of culture	0.113<br>(0.0928)	-0.0502<br>(0.106)	-0.159<br>(0.119)
The actual population	0.0504<br>(0.0342)	-0.159***<br>(0.0403)	-0.202***<br>(0.0460)
Proportion of elderly	-0.762**<br>(0.310)	1.087***<br>(0.386)	-0.153<br>(0.347)
Proportion of young adults	0.259<br>(0.182)	-0.0959<br>(0.214)	0.0133<br>(0.241)
Proportion of disability	-0.414<br>(0.360)	0.898*<br>(0.488)	0.0682<br>(0.337)
The proportion of subsistence allowances	0.0316<br>(0.160)	-0.926***<br>(0.188)	-0.723***<br>(0.190)
Agricultural land transfer-out	-0.225<br>(0.172)	0.274<br>(0.198)	0.0658<br>(0.226)
Agricultural land transfer-into	-0.248<br>(0.202)	-0.270<br>(0.231)	-0.233<br>(0.242)
Social networking	0.0407***<br>(0.0158)	-0.000426<br>(0.0177)	0.0226<br>(0.0233)
Competence training	0.124	-0.272**	-0.235

	(0.119)	(0.125)	(0.183)
Distance	-0.000431<br>(0.00119)	0.00387***<br>(0.00136)	0.00877***<br>(0.00159)
Instrumental variable	1.467***<br>(0.255)		
Constant term	-1.171**<br>(0.539)	9.326***<br>(0.568)	8.700***<br>(0.663)
ρ_1		-0.9554267<br>***<br>(0.022608)	
ρ_0		-0.0005695<br>(0.3034374)	
LR test of equation independence		32.5***	
Logarithmic likelihood value		-1395.9153	
Goodness of fit Wald test		101.43***	
Sample size		728	

4.2 The analysis on the treatment effect of microcredit for poverty-alleviated population on the income of farmers' production and operation

The estimation results of the treatment effect of farmers' access to microcredit for poverty-alleviated population on their production and operation income are as shown in table 3. The average treatment effect of farmers ' access to microcredit for poverty - alleviated population on their production and operation income is significantly positive at the statistical level of 1%. The estimation results of ATT show that under the counterfactual assumption, if farmers who obtain microcredit for poverty-alleviated population do not receive loans (counterfactual), their production and operation income will decrease from 8.028 to 7.717, a decrease of 3.87% (0.311/8.028) ; the estimation results of ATU show that under the counterfactual hypothesis, if the farmers who have not obtained the microcredit of the poverty-alleviated population get the loan (counterfactual), their production and operation income will rise from 7.794 to 9.969, an increase of 27.91% (2.175/7.794). According to this, it can be seen that the acquisition of micro-credit for the poverty-alleviated population has significantly increased the income of farmers ' production and operation. If the farmers who do not obtain micro-credit for the poverty-alleviated population are loaned, the income of their

production and operation income is more obvious, which can increase the income of farmers' production and operation by 3.87% ~ 27.91%.

Table 3. The average treatment effect of microcredit for poverty-alleviated population on farmers' production and operating income

**Group**	**Decision stage**		**Average treatment effect**	
	Loaned	Unloaned	ATT	ATU
Loaned Group （N=459）	8.028 （0.02）	7.717 （0.029）	0.311*** （0.033）	—
Unloaned group （N=269）	9.969 （0.032）	7.794 （0.038）	—	2.175*** （0.05）

In order to more clearly reflect the income effect of microcredit for poverty-alleviated population, the probability density distribution maps of production and operating income of farmers who have obtained and have not obtained microcredit for poverty-alleviated population are described respectively (Figure 1). The ATT of the left figure shows that if farmers who have obtained microcredit for poverty-alleviated population have not been loaned, the probability density distribution curve of their production and operating income is obviously shifted to the left. Lenders are in the 'counterfactual 'situation that they do not obtain microcredit for the poverty- alleviated population, the production and operating income of farmers is significantly reduced. The ATU of the right figure shows that the probability density distribution curve of the production and operation income of the uncredited households in the ' counterfactual ' situation of obtaining microcredit for the poverty-alleviated population has shifted significantly to the right, indicating that if the uncredited households obtain microcredit for the poverty-alleviated population, their production and operation income will increase significantly. Therefore, the research hypothesis 1 is verified.

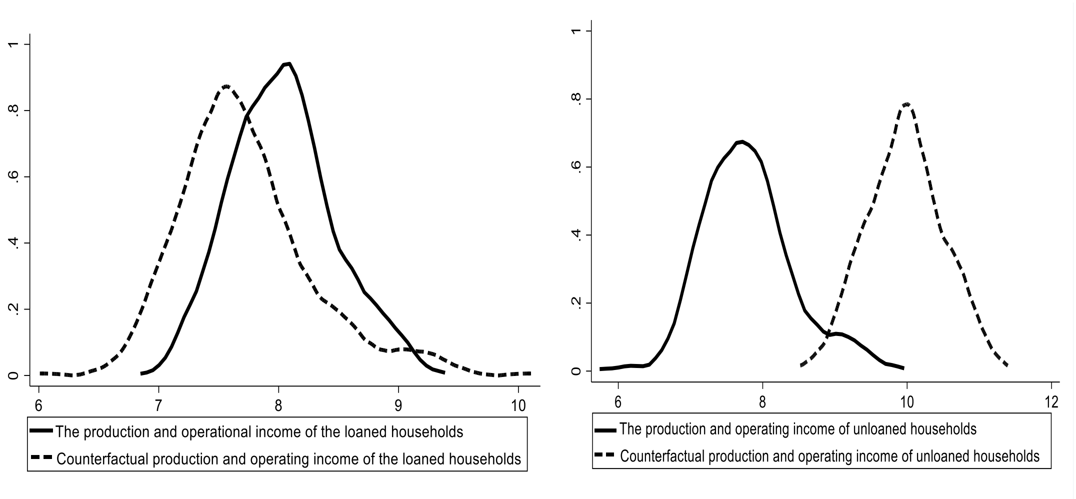

Fig1. Probability density of farmers' production and operation income in two cases

4.3 Robustness test

In order to verify the reliability of the above research conclusions, this paper uses three methods to test the robustness of the effect of income increase on the microcredit for poverty-alleviated population by replacing the empirical method, tail-down processing, and replacing the explanatory variables, and obtains table 4.

Robustness test method 1: Replace the empirical method. In this paper, the propensity score matching (PSM) method is used to estimate the effect of the microcredit for poverty-alleviated population on the income of farmers' production and operation. The farmers who obtained microcredit for the poverty-alleviated population were taken as the control group, and the farmers who did not obtain microcredit for poverty-alleviated population were taken as the treatment group, the nearest neighbor matching (K=4) is selected for matching. This part only reports the estimation results of the PSM model. The ATT value of the impact of microcredit for poverty-alleviated population on the income of farmers ' production and operation is 0.289, indicating that the microcredit for poverty-alleviated population has a significant effect on the income of farmers' production and operation, which is consistent with the estimation results of the ESR model. The difference is that the effect of microcredit for poverty-alleviated population on farmers' production and operation income obtained by PSM method is lower than that of ESR model. This is because the PSM model does not take into

account the influence of unobservable factors, resulting in biased estimation results. The ESR model not only solves the problem of self-selection and endogeneity of microcredit for the poverty-alleviated population, but also fully considers the selective bias caused by observable and unobservable factors. The bias term obtained in the first stage is automatically added to the second stage to estimate the impact of microcredit for the poverty-alleviated population on the income of farmers' production and operation, and the estimation results are more scientific [21].

Robustness test method 2: Tailing treatment. In order to eliminate the influence of extreme values of data on the estimation results [22], referring to the ideas of Falck et al. [23] and Pan et al. [24], the method of tail treatment is adopted to eliminate the extreme values of 5 % at the beginning and end of the data of farmers' production and operation income, and the model is estimated again. The ATT value of the impact of microcredit for poverty-alleviated population on farmers' production and operation income is 0.43, indicating that microcredit for poverty-alleviated population has a significant effect in promoting farmers' production and operation income, which is consistent with the above conclusions.

Robustness test method three: replace the explained variable. In fact, after many farmers obtain microcredit for the poverty-alleviated population, they put the assets such as cattle and sheep purchased by the loan into companies or cooperatives for dividends, and a small number of farmers put the credit funds into banks or lending enterprises to obtain interest income. This part of the income is also generated by microcredit for the poverty-alleviated population, but it is property income in income statistics. In order to further demonstrate the income effect of microcredit for the poverty-alleviated population, the property income and production and operation income are added to form property and production and operation income, and the property and production and operation income are replaced with new explanatory variables. The ATT value of the impact of microcredit for poverty-alleviated population on farmers' production and operation income is 0.383, indicating that microcredit for poverty-alleviated population has a significant role in promoting farmers' production and operation income, which is consistent with the above conclusions.

Table 4. Robustness test

Robustness test method	Loaned	Unloaned	ATT	t
Propensity score matching	8.011	7.722	0.289**	2.67
Tailing processing	8.038	7.608	0.43***	13.66
Replace the explained variable	8.106	7.723	0.383***	11.983

4.4 Heterogeneity analysis

Considering the heterogeneity of the effect of income increase on the microcredit for the poverty-alleviated population between the households who have lifted out of poverty and the monitoring objects. Therefore, we further study the differences in the production and operating income of the two types of households that have lifted out of poverty and the monitoring objects. As shown in Table 5, from the perspective of households that have lifted out of poverty, for the ATT of the loaned group, if the households that have lifted out of poverty who have obtained microcredit for the poverty-alleviated population are not loaned, their production and operating income will decrease from 8.143 to 7.9, a decrease of 2.98 % (0.243 / 8.143). For the ATU of the non-loaned group, if the households that have lifted out of poverty who have not obtained microcredit for the poverty-alleviated population are loaned, their production and operating income will increase from 7.856 to 10.003, an increase of 27.33% (2.147 /7.856). From the perspective of monitoring objects, for the ATT of the loaned group, if the monitoring objects of obtaining microcredit for the poverty-alleviated population are not loaned, their production and operation income will decrease from 7.933 to 7.514, a decrease of 5.28% (0.419/7.933). For the ATU of the non-credited group, if the monitoring object of the non-credited microcredit of the poverty-alleviated population is loaned, its production and operation income will increase from 7.753 to 10.107, an increase of 30.36% (2.354 /7.753). To sum up, the acquisition of microcredit for poverty-alleviated population by the households that have lifted out of poverty and the monitoring objects can significantly increase the income of production and operation, and the income increase effect of production and operation income of the

monitoring object is significantly higher than the households that have lifted out of poverty, which is 2.3 % higher.

Table 5. Differences in the average treatment effect of different types of farmers

Household type	Group	Decision stage		Average treatment effect	
		Loaned	Unloaned	ATT	ATU
Households out of poverty	Loaned group （N=218）	8.143 （0.036）	7.9 （0.043）	0.243*** （0.056）	—
	Unloaned group （N=106）	10.003 （0.06）	7.856 （0.064）	—	2.147*** （0.088）
Monitored object	Loaned group （N=241）	7.933 （0.026）	7.514 （0.037）	0.419*** （0.045）	—
	Unloaned group （N=163）	10.107 （0.04）	7.753 （0.051）	—	2.354*** （0.065）

4.5 The Analysis of difference in the effect of obtaining microcredit for the poverty-alleviated population on the production and operating income of farmers with different initial endowments.

Considering that the production and operation income of farmers may be skewed or abnormal, the model estimation of the impact of microcredit for poverty-alleviated population on the production and operation income of farmers will be biased. At the same time, the production and operation income of farmers is used as the basis for measuring the initial endowment of farmers' production and operation, which can show the impact of microcredit for poverty-alleviated population on the production and operation income from the whole distribution of production and operation income. Based on this, this study uses the quantile regression model to estimate the difference in the effect of microcredit for poverty-alleviated population on the production and operation income of farmers with different initial endowments. Drawing on the ideas of Li Yajuan and Ma Ji [25], this study classifies farmers with production and operating income at 10 %, 25 %, 50 %, 75 %, and 90 % quantiles as low, medium-low, medium, medium-high, and high initial endowment households, respectively.

As shown in the quantile regression results of Table 6, there is a significant difference in the impact of obtaining microcredit for poverty- alleviated populations on the production and operational income of farmers. At the 10 %, 25 %, 50 % and 75 % quantiles, the coefficients of the impact of obtaining microcredit for poverty- alleviated populations on the income of farmers ' production and operation are 0.305,0.389,0.296 and 0.229, respectively. The effect of increasing the income of production and operation income of farmers with low, medium and low initial endowments who obtain microcredit for poverty-alleviated population is greater than that of farmers with medium and high initial endowments. It is not difficult to see that the efficiency effect is decreasing as a whole, which can verify the research hypothesis 2. For small farmers, the credit funds invested in expanding the scale of production and operation have a marginal diminishing effect, so for the households that that have lifted out of poverty and monitoring objects with low initial endowments, the income-increasing effect of investment credit on farmers' production and operation income is greater than that of high initial endowments. That is to say, the microcredit for the poverty-alleviated population has a ' raising the low ' effect, and it also shows that the microcredit for the poverty-alleviated population has a relatively limited effect on the income increase of farmers with high initial endowments. In addition, at the 90 % quantile, the effect of obtaining microcredit for poverty alleviation population on the income increase of production and operation income of farmers with high initial endowment is not significant, and the coefficient is much smaller than other quantiles. The possible reason is that the production and operation scale of high initial endowment households is relatively large, and the role of microcredit for the poverty-alleviated population with a cap of 50,000 in expanding the scale of production and operation is relatively limited. It can be seen that in order to promote the production and operation income of poverty-alleviated households and monitoring objects with high initial endowment, it is necessary to seek other paths other than the microcredit policy for the poverty-alleviated population.

Table 6. Quantile regression of microcredit for poverty-alleviated population on farmers' production and operation income

Variable	Explained variable : The natural logarithm of per capita production and operation income				
	(1)	(2)	(3)	(4)	(5)
	q10	q25	q50	q75	q90
Microcredit for poverty-alleviated population	0.305*<br>(0.160)	0.389**<br>(0.151)	0.296**<br>(0.117)	0.229**<br>(0.107)	0.101<br>(0.118)
control variable	control	control	control	control	control
constant term	6.498***<br>(0.669)	7.941***<br>(0.666)	8.712***<br>(0.518)	9.307***<br>(0.518)	9.604***<br>(0.575)
R^2	0.1714	0.1433	0.1332	0.1141	0.1094
sample size	728	728	728	728	728

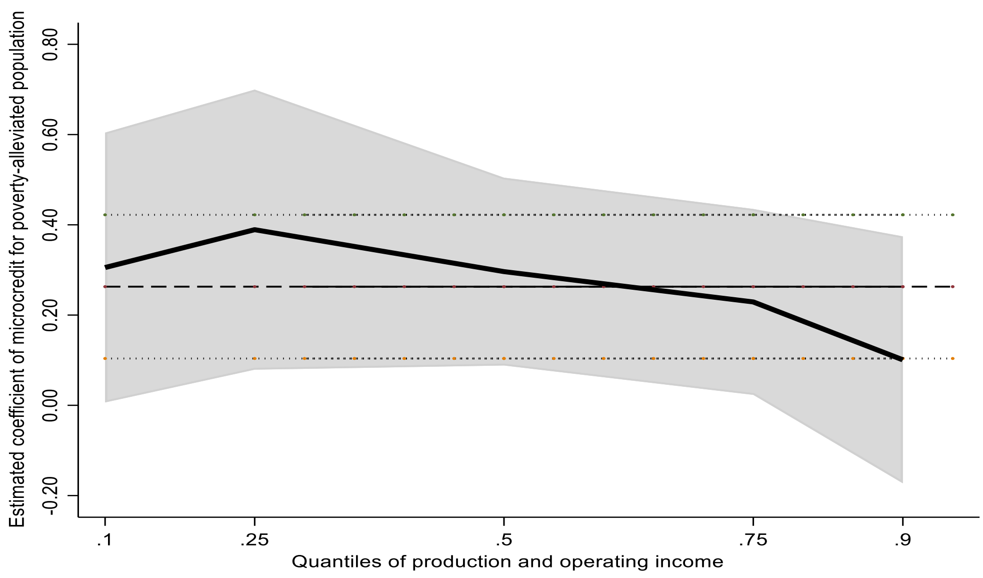

Fig 2. Full quantile regression coefficient and its change

Land is an important carrier for farmers to carry out production and operation activities, and it is also one of the main sources for farmers to obtain production and operation income. Therefore, according to the actual operating land area of farmers, this study divides the initial endowment situation into small-scale households with lower initial endowment of land (the actual operating area of land≤9) and large-scale households with higher initial endowment of land (the actual operating area of land> 9). The OLS model is used for grouping regression. The regression results are shown in table 7. When farmers are small-scale households, the estimated coefficient of the

impact of microcredit for poverty-alleviated population on their production and operating income is 0.312, and it is significant at the statistical level of 1%. It shows that for small-scale households with low initial endowment of land, their production and operation investment is relatively low, but the marginal income of production and operation investment is high. If they obtain credit funds, they can revitalize their production and operation activities under the existing production scale conditions and increase their income. When farmers are large-scale households, the impact of obtaining micro-credit for the poverty-alleviated population on their production and operation income is not significant and the coefficient is relatively small. There are two possible reasons. First, the production and operation investment of large-scale households is relatively large. In order to maintain or expand the scale of production and operation, financing through a limited amount of microcredit for the poverty-alleviated population may not meet the capital needs. Second, large-scale households are more likely to achieve economies of scale, and their production and operating income is relatively high, and they do not need to be financed by obtaining microcredit for poverty-alleviated population. This also verifies hypothesis 2, that is, microcredit for the poverty-alleviated population has a ' raising the low ' effect, and the acquisition of microcredit for the poverty-alleviated population has a higher income-increasing effect on farmers with low initial land endowment.

Table 7. Differences in the effect of microcredit on the income increase of poverty-alleviated population under different production scales

**Variable**	(1)<br>**Small-scale households（The actual operating area of land≤9)**	(2)<br>**Large-scale households (Actual operating area of land > 9)**
Microcredit for poverty - alleviated population	0.312***<br>(0.0927)	0.171<br>(0.172)
control variable	control	control
constant term	8.308***<br>(0.469)	8.474***<br>(0.767)
R2	0.1950	0.2622

sample size	563	165

5.Conclusions and implications

Based on the micro-survey data of 6 poverty-alleviated counties (cities) in a province of northwest China covering the households that have lifted out of poverty and monitoring objects, this study uses the endogenous transformation model to investigate the impact of microcredit poverty-alleviated population on the production and operation income of farmers and its heterogeneity. At the same time, the quantile regression model and OLS regression model are used to explore the difference in the effect of microcredit for poverty-alleviated population on the production and operation income of farmers with different initial resource endowments. The research results show that, first, the micro-credit of the poverty-alleviated population has an income-increasing effect. Specifically, under the counterfactual hypothesis, if the farmers who obtain the microcredit for the poverty-alleviated population are not loaned, their production and operation income will decrease by 3.87%, and if the farmers who do not obtain the microcredit for the poverty-alleviated population are loaned, their production and operation income will increase by 27.91%. After using the three robustness test methods of propensity score method (PSM), tail reduction and replacement of explanatory variables, the conclusion is still valid. In addition, both the households that have lifted out of poverty and the monitoring objects can significantly increase the income of production and operation, and the income-increasing effect of the monitoring objects is better than the households that have lifted out of poverty, that is, the income-increasing effect of obtaining microcredit for the poverty-alleviated population is greater than the households that have lifted out of poverty. Second, the microcredit for the poverty-alleviated population has a reducing effect. The income-increasing effect of the micro-credit of the poverty-alleviated population on farmers with low and medium-low initial endowments is greater than that of farmers with medium and medium-high initial endowments. At the same time, the micro-credit of the poverty-alleviated population has a high income-increasing effect on farmers with low land initial endowments. On the whole, the synergistic effect of the micro-credit

for the poverty-alleviated population on farmers with ' low→high ' initial resource endowments is decreasing as a whole, which also reflects that the microcredit for the poverty-alleviated population has a relatively limited income-increasing effect on farmers with high initial endowments.

The above research conclusions provide some empirical evidence for the effectiveness of financial precision assistance. Based on this, this paper draws the following enlightenment: First, maintain the smooth transition of the microcredit policy for the poverty-alleviated population during the period of consolidating and expanding the effective connection between the achievements of poverty alleviation and rural revitalization, and give full play to the ' hematopoietic ' function of inclusive finance. For the households that have lifted out of poverty and monitoring objects who have the conditions to develop production and operation, it is necessary to achieve full credit, and at the same time strengthen the supervision of the use of credit funds to ensure that the users of household borrowers are also accurately used for the development of production and operation. The second is to strengthen the ' internal ' accuracy of the micro-credit assistance policy for the poverty-alleviated population. In the long run, the micro-credit policy for the poverty-alleviated population should be tilted to the low-and middle-income monitoring objects and the households that have been lifted out of poverty. The inclusive credit policy for such high-income groups should gradually consider ' gradual retreat ' or gradually shift to the inclusive commercial loan model. The third is to strengthen the cultivation of credit loan awareness. On the one hand, from the perspective of moral integrity, loan households are constrained to standardize all credit funds for the development of production and operation, so as to play the ' hematopoietic ' role of microcredit for the poverty-alleviated population ; on the other hand, we should strengthen the publicity of the harmful consequences of overdue repayment, cultivate the awareness of loan borrowers to pay on time, and then improve the ' return ' rate of microcredit for the poverty-alleviated population. Fourth, timely and appropriate renewal or extension of loans should be carried out. For some people affected by natural disasters, large fluctuations in market prices and other factors, we should first consider reducing the repayment pressure of such households, and timely

and appropriate renewal or extension of loans should be carried out to avoid the risk of returning to poverty due to excessive repayment pressure.

Author Contributions:

Conceptualization: Yin Liu, Linchong Ma.

Methodology: Lu Fan.

Software: Yin Liu.

Formal analysis: Lu Fan.

Investigation: Yin Liu, Linchong Ma.

Resources: Yin Liu, Linchong Ma.

Data curation: Yin Liu.

Writing—original draft preparation: Yin Liu.

Writing—review and editing: Yin Liu, Lu Fan.

Visualization: Yin Liu.

Supervision: Lu Fan.

Project administration: Lu Fan.

Funding acquisition: Yin Liu, Linchong Ma, Lu Fan.

Funding: This research was funded Social Science Fund of Xinjiang Uygur Autonomous Region (20BJY051).

Competing Interests: The authors have declared that no competing interests exist.

References :

1. Lin WL, Ji XK. From Escaping from Absolute Poverty to Common Prosperity of Rural Households in Rural Areas. *Chinese Rural Economy*.2022; (08):2-15.
2. Koomson I , Villano R A , Hadley D . Effect of Financial Inclusion on Poverty and Vulnerability to Poverty: Evidence Using a Multidimensional Measure of Financial Inclusion. *Social Indicators Research: An International and Interdisciplinary Journal for Quality-of-Life Measurement*. 2020; 149.
3. Weng C, Zhu HG, Chen J. Can poverty alleviation microcredit improve the performance of farmers self-employment. Journal of Agrotechnical Economics.2021; (10):38-52.
4. Chen Z, Zhang J, Gong YX. A special investigation on the implementation of micro-credit poverty alleviation policy in Anhui Province. *Contemporary Economics*.2019; (06):73-78.
5. Jia JX, Qin C, Liu YZ. The policy design of ' top-down ' and ' bottom-up ' integration-based on the empirical analysis of rural development poverty alleviation projects. *Social Sciences in China*. 2017; (09):68-89+206-207.
6. Wang LJ, Zhou JJ, Chen KQ, et al. The Impact of Inclusive Finance on Multidimensional Poverty in Rural China. *Progress in Geography*.2022; 41(11):1991-2003.
7. Zhu X, Li ZN. The Economic Impact of Farm Credit：An Empirical Investigation based on IVQR Model. *Systems Engineering-Theory & Practice*.2007; (02):68-75.
8. Wang WC, Zhou JY. Analysis of the income effect of borrowing by different income groups in rural areas - Based on the survey data of farmers in rural northeast China. *Chinese Rural Economy*.2012; (05):77-84.
9. Lv YB, Zhang L, Wang Z. Analysis of regional differences in China 's rural financial development. *Statistics & Decision*.2012; (19):111-115.
10. Qi HQ, Zhang JX. Rural Inclusive Finance Development and the Sustainability of Poverty Reduction: Dual Perspectives Based on Multidimensional Relative Poverty and Poverty Vulnerability. *Inquiry into Economic Issues*. 2023; (07):158-175.
11. Cui QL, Wang CX. The Research on Common Prosperity Effect of Microcredit Development. *Journal of Technology Economics*. 2023; 42(07):141-151.

12. Zhou ML. Poverty alleviation, rural revitalization and supply-side reform of financial poverty alleviation. J*ournal of Southwest Minzu University (Humanities and Social Sciences Edition)* . 2020; 41(01):115-123.
13. Wu BJ, Ge YH, Ma JJ. Performance Comparison between Fiscal Poverty Alleviation and Financial Poverty Alleviation in the Period of Targeted Poverty Alleviation: From the Perspective of Heterogeneity of Poverty Alleviation and Multidimensional Poverty. *Chinese Rural Economy.* 2019; (07):21-36.
14. Yin ZC, Guo PY, Zhang LW. “Active Spring from the Source”：The Impact of Targeted Poverty Alleviation Policy on Household Credit in Rural China. *Journal of Management World.* 2020; 36(02):59-71+194+218.
15. Yan J, Qiang GL, Liu QJ. Poverty-alleviating Microcredit, Farmer Income, and Anti-poverty Performance. *Financial Economics Research.*2019; (4)32-44.
16. Li JH, Lu Q. The impact of digital finance on farmers' adoption of green production technologies. *Resources Science.* 2022; 44(12):2470-2486.
17. Ma WL, Renwick A, Nie P, et al. Off-farm work, smartphone use and household income: Evidence from rural China - ScienceDirect. *China Economic Review.* 2018; 52:80-94.
18. Huo Y, Xia WH, Lang YC, et al. The impact of green control technology adoption on cotton farmers’ welfare in Xinjiang. *Resources Science.* 2023; 45(01):130-143.
19. He J, Li QH. Digital Finance and Farmers' Entrepreneurship. *Chinese Rural Economy.* 2019; (01):112-126.
20. Yin ZC, Jiang JL, Song XW. The Impact of Social Network on Household Lending Behavior —An Empirical Study Based on China Household Finance Survey Data. *Journal of Northeast Normal University(Philosophy and Social Sciences)* .2023; (05):128-137.
21. Li CS, Liu XC. The Impacts of Land Rental Market Participation on Entrepreneurship: An Empirical Analysis Based on the Endogenous Switching Probit Model. *Chinese Rural Economy.* 2020; (05):96-112.
22. Liu EM, Huang J. Risk Preferences and Pesticide Use by Cotton Farmers in China. *Journal of Development Economics.*2013; 103: 202-215.
23. Falck O, Alfred LA, Ruhose J. Cultural Biases in Migration: Estimating Non－monetary Migration Costs. *Papers in Regional Science.* 2016; 2(04) : 1-28.

24. Pan D, Lu Y, Kong FB. The Impact of Participation Degree and Time of Returning Farmland to Forest on Farmers' Household Income: Empirical Analysis Based on a Multiple Endogenous Regression Model. *Journal of Agrotechnical Economics*. 2022; (06):19-32.
25. Li YJ, Ma J. Analysis of Income Effect Differences of Scientific Fertilization Technology—An Empirical Estimation Based on farmers' Initial Endowment. Journal of Agrotechnical Economics.2021(07):18-32.
