# Supplementary material for: Does microcredit for the poverty-alleviated population have an income-increasing effect? Also on the “Raising the Low” effect
Source: PLoS One. 2025 Aug 1;20(8):e0327431. doi: 10.1371/journal.pone.0327431 (PMC12316256; doi:10.1371/journal.pone.0327431)
Supplement: Attachment — Submitted filename: response2(0926).doc [file pone.0327431.s004.doc]

Dear Reviewer :

Hello ! Thank you very much for taking the time to review our paper, and put forward valuable amendments. In view of your questions and suggestions, we have carried on the careful analysis and discussion, and the paper has carried on the corresponding modification and improvement. The following is our specific response to your review comments :

**Revision suggestion1:** Title is somewhat confusion. Poverty alleviated population?? What do you mean by it and clearly explain it in the beginning of introduction. I think title should be changed as well.

**Reply：**This paper mainly studies the impact of ' micro-credit policy for poverty-alleviated population on farmers ' production and operating income. Microcredit for poverty-alleviated population is a preferential financial policy (mortgage-free and guarantee-free, financial full discount ), provided by China for low-income people ( the households that have lifted out of poverty and monitoring objects ). For ease of understanding, the article adds criteria for distinguishing different types of households in rural China and a schematic diagram of China 's support policy system.

**Revision suggestion2:**.Introduction should be rewritten and discuss the importance of the research topic, clear research gap, objectives and study contribution with adequate literature support not merely collection of words, sentences, and fluency.

**Reply：**The article further presents the introduction and literature review as two parts. In the literature review part, it clarifies the existing research viewpoints and deficiencies as well as the contribution of this study.

**Revision suggestion3：**Review of literature section is missing.

**Reply：**The introduction of the original article contains a literature review. In order to make the structure more reasonable, the introduction and literature have been divided into two parts.

**Revision suggestion4：**Manuscript needs rearrangement of section as well. Econometric methods should be placed after the data collection and hypothesis part.

**Reply：**The structure of the article has been adjusted according to the opinions.

**Revision suggestion 5：**.Sampling procedure is in-adequality explained. Authors should rewrite this whole section related to data collection and explain which sampling technique did they use in data collection. What was their sampling farm and what measures did they take to collect a representative sample. Moreover, authors should also clearly explain the reason behind selecting specific study region.

**Reply：**Taking into account the length and focus of the paper, we have made the following additions and explanations to the sampling procedure, sample selection, and study area : The data in this paper are derived from the sample survey data of farmers in six poverty-alleviated counties (cities) in Hotan Prefecture, Xinjiang Uygur Autonomous Region, in January 2023. Hotan Prefecture is one of the areas where poverty alleviation is difficult and the scale of poverty alleviation is large during the period of poverty alleviation in China. It is typical to study the income effect of microcredit for poverty-alleviated population in the region under the background of comprehensively promoting rural revitalization. Based on the comprehensive factors such as geographical location, economic development level, number of monitoring objects, and village attributes, this survey randomly selected 10 villages from 5 townships in each of the six poverty-alleviated counties (cities). Among them, each county (city) extracts 8 poverty-alleviated villages (including large and medium-sized settlements) and 2 general villages with relatively heavy rural revitalization tasks, and each township extracts 2 villages ( including large and medium-sized settlements ). According to the roster of farmers provided by the sampling village, considering the family income status, health status, disability and so on, a random sampling survey was carried out in the sampling village. This paper mainly focuses on the survey of he households that have lifted out of poverty, monitoring objects and general households with low income, collecting and mastering the income status, assistance status, micro-credit, industrial development and rural governance and construction of the survey objects. A total of 1350 questionnaires were collected. According to the policy, the borrowing objects of microcredit for the poverty-alleviated population must be poverty-alleviated households and monitoring objects, and must be used for the development of production and operation. Therefore, this paper excludes general households and farmers without production and operation income. There are 738 valid samples, of which 329 households have been lifted out of poverty (excluding monitoring objects ). The monitoring objects are 409 households ( including 54 households with sudden serious difficulties, 183 households with unstable poverty alleviation, and 172 households with marginal poverty-prone).

**Revision suggestion 6：**.Questionnaire used for cross sectional survey must be explained in a separate subsection. I think authors should have three clear subsection of study area selection, survey instrument, sampling procedure and data collection.

**Reply：**When doing descriptive statistics, the article has explained the content of the questionnaire involved. Questions such as the selection of study areas, survey instrument, sampling procedures and data collection have been answered in question 5.

**Revision suggestion 7：**Result section needs a better organization only study findings according to the study objective must be provided in this section. Supporting material should be in the in supplementary section or appendix.

**Reply：**The article supplements the discussion part and explores from three aspects : research conclusions, research discussions, and countermeasures.

**Revision suggestion 8：**.Discussion section should be separated from the results and literature support must be provided the study findings.

**Reply：**The research conclusions and discussions have been separated. And supplemented the literature support.

**Revision suggestion 9:** Limitation of study is missing.

**Reply：**In the research discussion section, relevant content on the limitations of the research was added.

**Revision suggestion 10:** Policy recommendation section is needed.

**Reply：**The part of the policy recommendation has been presented separately as a title, and the relevant content has been improved.

**Revision suggestion 11:**More Literary support must be provided throughout the manuscript.

**Reply：**Since most of the content in the article is based on empirical analysis results, no more literature is cited and added.

**Revision suggestion 12:**.Language of the manuscript is not good enough and must be improved.

**Reply:**The language expression of some contents has been adjusted and modified.

The above is a response to your review comments. In addition, it needs to be further explained that considering that the sample is more reasonable, the revised draft eliminates some variables that are missing more, such as the total number of household population, which increases the total number of samples to 738. The large sample size makes the research more convincing. Although the corresponding regression results have also changed, the overall conclusion has not changed much. At the same time, this rework has attached a full set of original data and code.

Here, we will resubmit the revised paper to you, please review it again. If you have any other questions or need further discussion, you can contact us at any time. Finally, thank you again for your review and guidance of our manuscripts, and look forward to your review feedback.

Thank you and kind regards.

Yours sincerely，

Lu Fan，PhD
