# Supplementary material for: Does microcredit for the poverty-alleviated population have an income-increasing effect? Also on the “Raising the Low” effect
Source: PLoS One. 2025 Aug 1;20(8):e0327431. doi: 10.1371/journal.pone.0327431 (PMC12316256; doi:10.1371/journal.pone.0327431)
Supplement: Attachment — Submitted filename: response2.doc [file pone.0327431.s005.doc]

Dear Reviewer :

Hello ! Thank you very much for taking the time to review our paper, and put forward valuable amendments. In view of your questions and suggestions, we have carried on the careful analysis and discussion, and the paper has carried on the corresponding modification and improvement. The following is our specific response to your review comments :

**Revision suggestion1:** **General comments.** （1）“Raising the Low" This need to come out clearly across the paper – from abstract to conclusion. （2）In all Tables, remove the vertical lines in all tables.

**Reply：**（1）It has supplemented the relevant explanation of the " Raising the Low " effect from the abstract to the conclusion, and explained why microcredit for the poverty-alleviated population has a " Raising the Low " effect from the perspectives of risk and return matching, as well as differences in funding needs and uses. （2）Vertical lines have been removed from all tables in the text.

**Revision suggestion2:Abstract.**（1）The policy recommendations are too broad, for instant, you recommend the need to promote the microcredit policy of the poverty-alleviated population from the aspects of policy stability and implementation precision. How and by doing what?（2）What policy can the study recommend for the for different categories of poverty-alleviated population.

**Reply：**（1）In this paper, the two mechanisms of physical capital investment and social capital are added. Therefore, in terms of suggestions, the following contents are added : we should increase the support for the production and operation of material capital investment of lenders, help them purchase means of production, equipment and technology, improve production efficiency, and at the same time expand the social capital of lenders, enhance their ability to obtain information and resources, and promote the increase of production and operation income.（2）The microcredit policy for the poverty- alleviated population is only aimed at the households that have lifted out of poverty and the monitoring objects who are willing to lend and have the ability to produce and operate. In the process of policy implementation, it is clearly stated that loans should be provided to all who need them. However, due to the low awareness of the policy among the households, there are still a large number of people who have not taken out loans. Considering that the microcredit policy for the poverty-alleviated population has a better income-increasing effect on the monitoring objects than the households that have lifted out of poverty, it is added in the policy enlightenment. ' For the uncredited households, especially the monitoring objects, we should strengthen policy publicity and guidance, ' emphasizing that the policy tendency should pay attention to the monitoring objects. In addition, this study finds that microfinance for the poverty-stricken population has a higher income-increasing effect on farmers with low-income initial endowment, that is, it has a ' raising the low ' effect. Therefore, in the policy recommendations, it is proposed to strengthen the 'internal' accuracy of the microcredit policy for the poverty- alleviated population, and tilt to the monitoring objects with low-income initial endowments and households that have been lifted out of poverty. The inclusive credit policy for groups with high -income initial endowments should gradually consider 'gradual regression' or gradually shift to the inclusive commercial loan model.

**Revision suggestion3: Introduction.** （1）The paragraph is too long split it. （2）Discuss the novelty of the paper. The statement, “it is of great practical significance to study the income increase effect and raising the low effect of microcredit for the poverty-alleviated population” is not adequate. (Page 2 last sentence)

**Reply：**（1）The paragraph has been divided into two paragraphs.（2）Studying whether the microcredit for the poverty-alleviated population has an income-increasing effect, how effective is its implementation, and what is the mechanism of action ? Can we achieve the goal of 'raising the low' and 'expanding the middle' ? It is of great practical significance in promoting rural revitalization and achieving common prosperity.

**Revision suggestion4:** **Literature review. （**1）In page 4, you refer to the period of poverty alleviation; which period is this? Has the period ended or is China still under the transition period of poverty alleviation?（2） So why is microcredit segmentation analysis for different categories of poverty-alleviated population important?

**Reply：**（1）2020 and before is the period of poverty alleviation. Since absolute poverty has been eliminated by various unconventional means at the end of 2020, in order to ensure that there is no large-scale return to poverty, 2021-2025 is called the transition period, that is, the transition from consolidating the achievements of poverty alleviation to comprehensive rural revitalization.（2）Since the beneficiary group of the previous poverty alleviation microfinance can only be identified as a group of poor households, but starting in 2020, the monitoring object ( Marginal poverty-prone households: this group is non-poor households ) is also included in the group enjoying microfinance. However, the existing research on the microcredit for poverty-alleviated population does not focus on such households, and still only focuses on the households that have lifted out of poverty (formerly poor households ). Therefore, comparing the differences between the two groups of households who have lifted out of poverty and the monitoring objects can not only enrich the existing research results, but also comprehensively evaluate the policy effect of microcredit for China 's poverty-alleviated population.

**Revision suggestion5:** **Descriptive results.** I was expecting to see the descriptive result for different categories of poverty-alleviated population (Table 3)

**Reply：**The descriptive statistical results of the two types of the households that have lifted out of poverty and the monitoring objects have been supplemented, which can be seen in the text.

**Revision suggestion6:** Empirical Test and Result Analysis. While introducing 5.1.1 (Point out that it explains the selection / decision equation) and for the introductory part of 5.1.2 (Point out that it explains the result / outcome equation. Though the results section is separated from the discussion, it is too long. The study needs to highlight major findings.

**Reply：**Although this part is not the focus of this study, in order to study the coherence and integrity, we use the same influencing factors to analyze what factors affect farmers ' access to microcredit policy for the poverty-alleviated population and what factors will affect farmers' production and operation income. We believe that this part is indispensable, so a lot of analysis has been carried out.

**Revision suggestion7:** Conclusion and Discussion.（1）Draw the conclusions must be drawn appropriately based on the data presented. Conclusions need to be based on objectives and major findings and provide policy implications based on the findings.（2）Why is the discussion presented after conclusion (Section 6.2), this is confusing to the reader.

**Reply：**（1）We add data description in the conclusion part, and enrich the policy implications based on the conclusion and survey results. See the text for specific modifications.（2）The conclusion part presents the specific research conclusions of this paper, which is the summary of the research results, and the discussion is the reflection and extension of the results. Therefore, this paper puts the discussion behind the conclusion to make the whole research system more complete.

**Revision suggestion8**: **Reference.** Format reference listed consistently according to PLOS ONE author guidelines.
 **Reply：**References have been adjusted according to journal requirements.

The above is a response to your review comments. Here, we will resubmit the revised paper to you, please review it again. If you have any other questions or need further discussion, you can contact us at any time. Thank you again for your review and guidance of our manuscripts, and look forward to your review feedback.

Thank you and kind regards.

Yours sincerely，

Lu Fan，PhD
